# Long non-coding RNA DLGAP1-AS1 facilitates tumorigenesis and epithelial–mesenchymal transition in hepatocellular carcinoma via the feedback loop of miR-26a/b-5p/IL-6/JAK2/STAT3 and Wnt/β-catenin pathway

**DOI:** 10.1038/s41419-019-2188-7

**Published:** 2020-01-16

**Authors:** Ye Lin, Zhixiang Jian, Haosheng Jin, Xiangling Wei, Xiongfeng Zou, Renguo Guan, Jianfeng Huang

**Affiliations:** Department of General Surgery, Guangdong Provincial People’s Hospital, Guangdong Academy of Medical Sciences, 106 Zhongshan Er Road, Guangzhou, 510080 China

**Keywords:** Cancer, Cell biology

## Abstract

Hepatocellular carcinoma (HCC) is one of the most common and lethal malignancies worldwide, and epithelial–mesenchymal transition (EMT) is a crucial factor affecting HCC progression and metastasis. Long noncoding RNAs (lncRNAs) have been validated to act as critical regulators of biological processes in various tumors. Herein, we attempted to elucidate the uncharacterized function and mechanism of lncRNA DLGAP1-AS1 in regulating tumorigenesis and EMT of HCC. In our study, DLGAP1-AS1 was shown to be upregulated in HCC cell lines and capable to promote HCC progression and EMT. Besides, DLGAP1-AS1 was proven to serve as a molecular sponge to sequester the HCC-inhibitory miRNAs, miR-26a-5p and miR-26b-5p, thus enhancing the level of an oncogenic cytokine IL-6, which could activate JAK2/STAT3 signaling pathway and reciprocally elevate the transcriptional activity of DLGAP1-AS1, thus forming a positive feedback loop. Moreover, we elaborated that the cancerogenic effects of DLGAP1-AS1 in HCC cells could be effectuated via activating Wnt/β-catenin pathway by positively regulating CDK8 and LRP6, downstream genes of miR-26a/b-5p. In conclusion, our results demonstrated the detailed molecular mechanism of DLGAP1-AS1 in facilitating HCC progression and EMT in vitro and in vivo, and suggested the potentiality of DLGAP1-AS1 as a therapeutic target for HCC.

## Introduction

Hepatocellular carcinoma (HCC), which is known as the most prevalent (75–85%) type of liver cancer, is a severe malignant tumor torturing patients from all over the world^1^. HCC is ranked the sixth most common cause of neoplasm and the third most frequent cause of cancer mortality worldwide^2^. Although numerous progress on surgical and medical techniques for HCC treatment have been made, the prognosis for HCC patients still remains poor with an overall 5-year survival rate of 5% approximately, largely owing to lack of more effective therapeutic methods, delayed diagnosis, as well as high rates of postoperative recurrence and metastasis^3,4^. Therefore, it is of considerable importance to elucidate underlying molecular mechanisms in relation to HCC progression to exploit novel therapeutic strategies.

Epithelial–mesenchymal transition (EMT) is characterized as a crucial biological process by which cells lose their epithelial features and acquire properties for migration and invasion^5^. In HCC, specifically, EMT has been proven to be crucial in determining tumor progression and metastasis, and can be accelerated by various biological factors, such as inflammatory cytokine interleukin 6 (IL-6), JAK2/STAT3 signaling, and dysregulation of Wnt/β-catenin pathway^6,7^. Therefore, our research principally focused on mechanisms to trigger EMT process of HCC cells in order to search for appropriate therapeutic approaches.

Long non-coding RNAs (lncRNAs) have been engaging great interest of scientific researchers. Basically, lncRNAs are classified as a sort of RNA transcripts containing more than 200 nucleotides in length with poor or no protein-coding ability^8,9^. Recently, it has been verified by accumulating evidence that lncRNAs are playing remarkable roles in regulating the multifarious processes of many diseases, including cancers such as HCC^10,11^. For instance, researchers have made numerous discoveries in recent years to disclose that various lncRNAs, such as TSLNC8, HNF1A-AS1, and PTTG3P, display aberrant expressions in HCC, and can act as tumor suppressors or oncogenes to regulate HCC progression and metastasis^12,13^.

In this study, we investigated the function and mechanism of the lncRNA named “discs, large (Drosophila) homolog-associated protein 1 antisense RNA 1”, or DLGAP1-AS1 for short, whose involvement in HCC remains uncharacterized. The results of our study demonstrated the participation of DLGAP1-AS1 in regulating tumorigenesis and metastasis of HCC in vitro and in vivo, and suggested that DLGAP1-AS1 could be a potential target for the treatment of HCC.

## Materials and methods

### Tissues specimen

A total of 60 primary HCC tissue samples and adjacent normal tissues were collected at Guangdong Provincial People’s Hospital. This study was approved by the Research Ethics Committee of Guangdong Provincial People’s Hospital. Written informed consents were obtained from all patients. Patients participating in this research did not receive treatment before surgery, chemotherapy or radiotherapy. The tumor samples were immediately frozen in liquid nitrogen and then kept at −80 °C.

### Cell culture and treatment

Normal liver cell (THLE-3), human HCC cells (SNU-387, Hep3B, SNU-182 and HepG2), and human embryonic kidney cell (HEK-293T) were obtained from American Type Culture Collection (ATCC; Manassas, VA, USA). Cells were cultured following the previous description^14,15^. Human recombinant IL-6, JAK2/STAT3 pathway inhibitor Cucurbitacin I and Wnt/β-catenin pathway activator CHIR99021 were all obtained from Sigma-Aldrich (St. Louis, MO, USA).

### Cell transfection

Specific small interfering RNAs (siRNAs) against DLGAP1-AS1 (si-DLGAP1-AS1#1, si-DLGAP1-AS1#2 and si-DLGAP1-AS1#3), negative control (si-NC) along with the pcDNA3.1 vector targeting DLGAP1-AS1, STAT3, CDK8 or LRP6 and the empty vector, were all acquired from Genechem (Shanghai, China). Besides, miR-26a/b-5p mimics, miR-26a/b-5p inhibitors, and NC mimics, NC inhibitors were from GenePharma (Shanghai, China). HepG2 or SNU-387 cells were separately transfected with these plasmids using Lipofectamine 3000 (Invitrogen, Carlsbad, CA, USA).

### Quantitative real-time PCR (qRT-PCR) analysis

For isolation of total RNA from cells, TRIzol reagent (Invitrogen) was employed in line with the supplier's protocol. Afterward, the reverse transcription was carried out with total RNA applying Transcriptor First Strand cDNA Synthesis Kit (Roche, Mannheim, Germany). qRT-PCR was implemented with SYBR Green I Master (Roche) on the LightCycler® 480 System (Roche). Relative gene level was normalized to GAPDH or U6 and expression fold change was calculated using the 2^−ΔΔCt^ method.

### CCK-8 assay

Cell proliferation was measured via Cell Counting Kit-8 (CCK-8) assay. Briefly, Transfected HepG2 or SNU-387 cells were transplanted in 96-well plates. CCK-8 solution (Sigma-Aldrich) was added to each well at 0, 24, 48, 72, and 96 h, respectively, and then cells were incubated for 3 h. Absorbance was measured at 450 nm with a microplate reader (BioTek, Winooski, VT, USA).

### Wound healing assay

Transfected HepG2 or SNU-387 cells were cultured till confluence was up to greater than 90%. Then, cell layers were scratched utilizing a plastic scriber. Upon that, cells were washed twice by phosphate buffer saline (PBS; Sigma-Aldrich) and incubated for 36 h. The wound was visualized and images were taken at 0 and 36 h with the inverted microscope (Nikon, Tokyo, Japan).

### TUNEL assay

Cell apoptosis was detected by terminal deoxynucleotidyl transferase-dUTP nick end labeling (TUNEL) assay. Transfected HepG2 or SNU-387 cells were washed by the use of PBS, followed by fixation with 1% paraformaldehyde (Sigma-Aldrich). TUNEL reagents (Millipore, Burlington, MA, USA) were applied for staining apoptotic cells, while cell nuclei were visualized using 4′,6-diamidino-2-phenylindole (DAPI) staining (Invitrogen). Optical microscopy (Olympus, Tokyo, Japan) was finally applied for analyzing.

### Western blot (WB) analysis

On the basis of previous description, western blot was carried out^16^. Following primary antibodies were bought from Abcam (Cambridge, UK): anti-E-cadherin (ab1416), anti-N-cadherin (ab18203), anti-Vimentin (ab8978), anti-Twist (ab175430), anti-JAK2 (ab108596), anti-p-JAK2 (ab32101), anti-STAT3 (ab119352), anti-p-STAT3 (ab32143), anti-CDK8 (ab224828), anti-LRP6 (ab75358), anti-β-catenin (ab32572), anti-p-β-catenin (ab27798), anti-Cyclin D1 (ab16663), anti-c-Myc (ab32072), anti-MMP9 (ab38898) and anti-GAPDH (ab8245).

### RNA immunoprecipitation (RIP) assay

RIP were implemented by employing a Magna RIP^™^ RNA-Binding Protein Immunoprecipitation Kit (Millipore). Ago2 antibody or IgG antibody was purchased from Abcam. Precipitated RNAs were subjected to qRT-PCR.

### RNA pull-down assay

The miRNAs were individually labeled with biotin through a Pierce RNA 3′ End Desthiobiotinylation Kit (Thermo Fisher Scientific, Waltham, MA). Bio-miR-26a-5p-WT/Mut or Bio-miR-26b-5p-WT/Mut was incubated with HepG2 or SNU-387 cell lysates adding magnetic beads (Invitrogen). After washing, qRT-PCR was conducted for the eluted pulled-down complex.

### Luciferase reporter assay

The wild-type or mutant interacting sequences of miR-26a/b-5p in DLGAP1-AS1 sequence or in 3′-UTRs of IL-6, CDK8, and LRP6 were subcloned into the pmirGLO dual-luciferase plasmid (Promega, Madison, WI, USA). They were named as DLGAP1-AS1-WT/Mut, IL-6-3′-UTR-WT/Mut, CDK8-3′-UTR-WT/Mut, and LRP6-3′-UTR-WT/Mut. These vectors were co-transfected into HEK-293T cells with indicated transfection plasmids. After 48 h of co-transfection, relative luciferase activities were examined utilizing dual-luciferase reporter assay system (Promega).

### Chromatin immunoprecipitation (ChIP) assay

After sonication of chromatin into fragments (500 bp), immunoprecipitation was carried out by adopting anti-STAT3 antibody or anti-IgG antibody. Precipitated DNA fragments were eventually extracted and subjected to qRT-PCR.

### TOP/FOP flash assay

HepG2 cells were treated with various transfection plasmids and TOP/FOP Flash plasmids (Upstate Biotechnology, Lake Placid, NY, USA). Relative luciferase activities were examined utilizing dual-luciferase reporter assay system (Promega).

### Xenograft in vivo analysis

The animal experiments were approved by the Ethics Committee of Guangdong Provincial People’s Hospital, according to the Guide for the Care and Use of Laboratory Animals by the National Institute of Health. Four-week-old BALB/c nude mice were purchased from Shanghai SLAC Laboratory Animal (Shanghai, China). Transfected HCC cells were injected into mice subcutaneously. Five mice in each group were measured. The tumor volume was measured every 4 days. After 4 weeks, mice were sacrificed and tumors were weighed.

### In vivo metastasis assay

In vivo metastasis assay was conducted as previously described^17^. Four-week-old SCID-Beige female mice were provided by the medical college of Guangdong Provincial People’s Hospital. All animal experiments were performed and finished in accordance with protocols provided by the Institutional Animal Care and Use Committee of Guangdong Provincial People’s Hospital.

### Enzyme-linked immunosorbent assay (ELISA)

Relative expression of IL-6 was detected with the ELISA kit (NeoBioscience, Shenzhen, China). Optical density was read with the microplate reader Victor X3 (PerkinElmer, Waltham, MA, USA) at 450 nm.

### Bioinformatics analysis

Tissue-specific or tumor-specific expressional pattern of DLGAP1-AS1 was obtained from UCSC Genome Browser online database (https://genome.ucsc.edu) and GTEx was displayed using the online database of Gene Expression Profiling Interactive Analysis (GEPIA; http://gepia.cancer-pku.cn). Interaction and binding site between RNAs were identified by starBase v3.0 online database (http://starbase.sysu.edu.cn). Transcription factor binding sites within DLGAP1-AS1 promoter were identified using JASPAR database (http://jaspar.genereg.net). MicroRNA (miRNA) family conservation and annotation confidence were evaluated by use of miRBase (http://www.mirbase.org) and TargetScan (http://www.targetscan.org/vert_72).

### Statistical analysis

Results were acquired from assays implemented thrice and presented as mean ± SD. *P* < 0.05 was considered statistically significant. Variance analyses were conducted via Student's *t*-test or one-way ANOVA. Statistical analyses were carried out by the use of SPSS 22.0 (IBM, Armonk, NY, USA). Pearson correlation test was applied for analyzing expression correlation.

## Results

### Upregulation of DLGAP1-AS1 was correlated with tumorigenesis of HCC

**Fig. 1:**
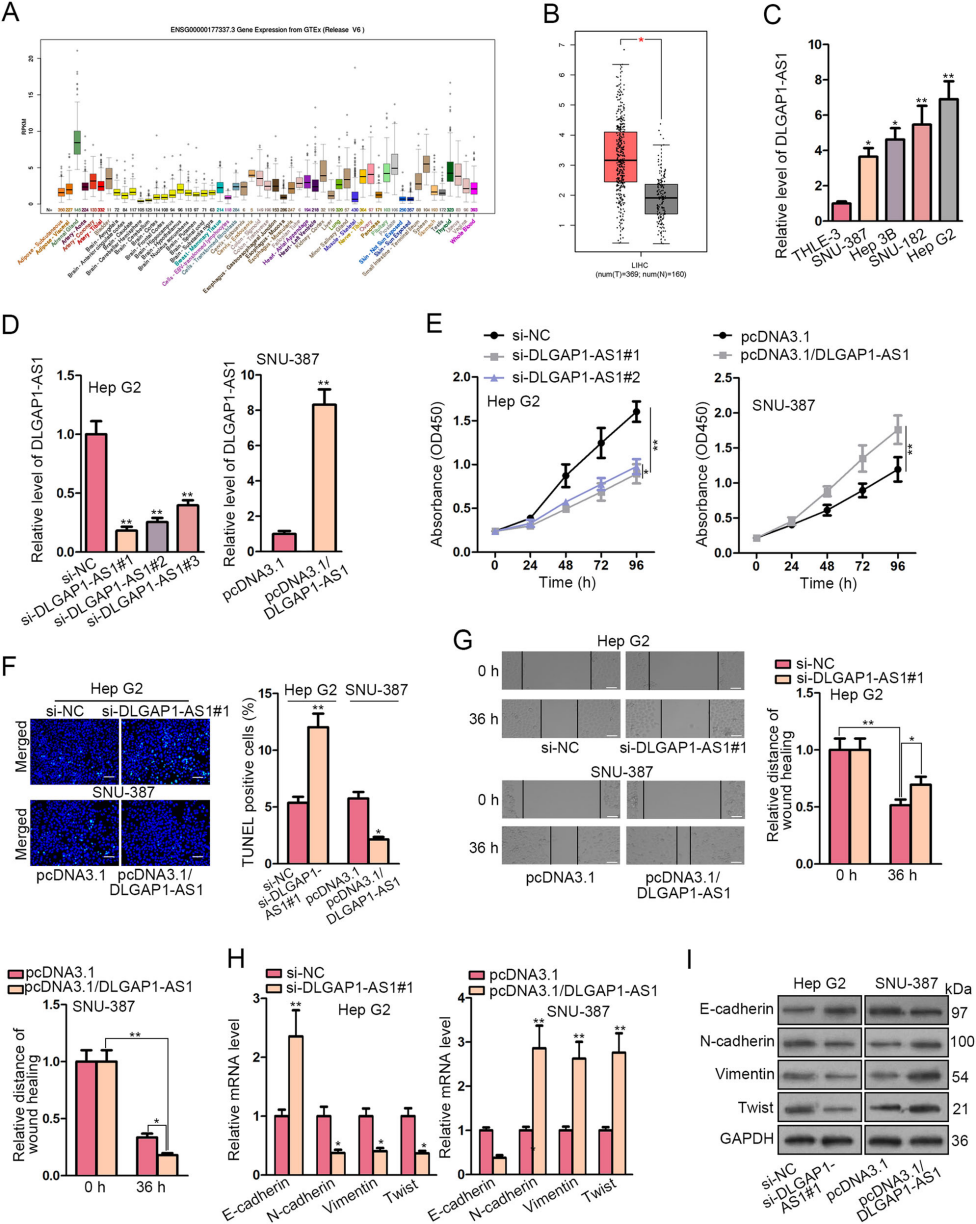
Upregulation of DLGAP1-AS1 was correlated with tumorigenesis of HCC. **a** DLGAP1-AS1 expression in normal human tissues (*n* = 570) were displayed by UCSC Genome Browser. **b** DLGAP1-AS1 expression levels in LIHC (red; *n* = 369) and normal (gray; *n* = 160) datasets obtained from GEPIA boxplot analysis. **c** DLGAP1-AS1 expression levels were assessed using qRT-PCR in four HCC cell lines and normal human liver epithelial cells THLE-3. **d** DLGAP1-AS1 knockdown and overexpression efficiencies were evaluated using qRT-PCR. **e**, **f** CCK-8 assay and TUNEL assay assessed the influence of DLGAP1-AS1 knockdown or overexpression on proliferation or apoptosis of Hep G2 and SNU-387 cells. Scale bar = 200 μm. **g** Wound healing assay was performed to determine the effect of DLGAP1-AS1 on HCC cell migration. Scale bar = 200 μm. **h**, **i** EMT-related factors in Hep G2 or SNU-387 cells after DLGAP1-AS1 knockdown or overexpression were respectively detected using qRT-PCR and WB. All data are presented as the mean ± SD of three independent experiments. *p < 0.05, **p < 0.01.

First of all, according to UCSC Genome Browser online database, DLGAP1-AS1 expression level was relatively low in normal human liver tissues (Fig. 1a). Comparatively, the significantly elevated DLGAP1-AS1 expression in liver hepatocellular carcinoma (LIHC) dataset in comparison with normal dataset was presented using GEPIA online database (Fig. 1b). Subsequently, DLGAP1-AS1 expression is upregulated in four individual HCC cell lines (Hep G2, SNU-182, Hep 3B, and SNU-387) in comparison with normal human liver epithelial cells THLE-3, where Hep G2 showed the highest level and SNU-387 showed the lowest level of DLGAP1-AS1 expression among these HCC cell lines, consistent with the results from bioinformatics analyses (Fig. 1c). Therefore, we established knockdown and overexpression models for DLGAP1-AS1 in HCC cell lines, respectively by transfecting DLGAP1-AS1 siRNAs into Hep G2 cells, and DLGAP1-AS1 overexpression plasmid into SNU-387 cells (Fig. 1d).

CCK-8 assay illustrated that the proliferation rate of Hep G2 cells transfected with DLGAP1-AS1 siRNAs was decreased compared with the si-NC group, while the proliferation rate of SNU-387 cells was increased in pcDNA3.1/DLGAP1-AS1 group compared with the vector control (Fig. 1e). As for cell apoptosis, TUNEL assay showed that DLGAP1-AS1 knockdown enhanced the apoptotic level of Hep G2 cells, whereas DLGAP1-AS1 overexpression reduced apoptosis in SNU-387 cells (Fig. 1f). Besides, wound healing assay illustrated that DLGAP1-AS1 knockdown reduced, while DLGAP1-AS1 overexpression enhanced the migration ability of HCC cells (Fig. 1g). In order to explore whether DLGAP1-AS1 could promote EMT process, we measured the levels of several representative EMT markers, and found that the mRNA and protein levels of the epithelial marker E-cadherin were raised by DLGAP1-AS1 knockdown and reduced by DLGAP1-AS1 overexpression, whereas the levels of the mesenchymal markers N-cadherin, Vimentin, and Twist showed the opposite tendency (Fig. 1h, l). These results suggested that DLGAP1-AS1 was closely correlated with tumorigenesis of HCC.

### DLGAP1-AS1 acted as a molecular sponge for miR-26a-5p and miR-26b-5p

**Fig. 2:**
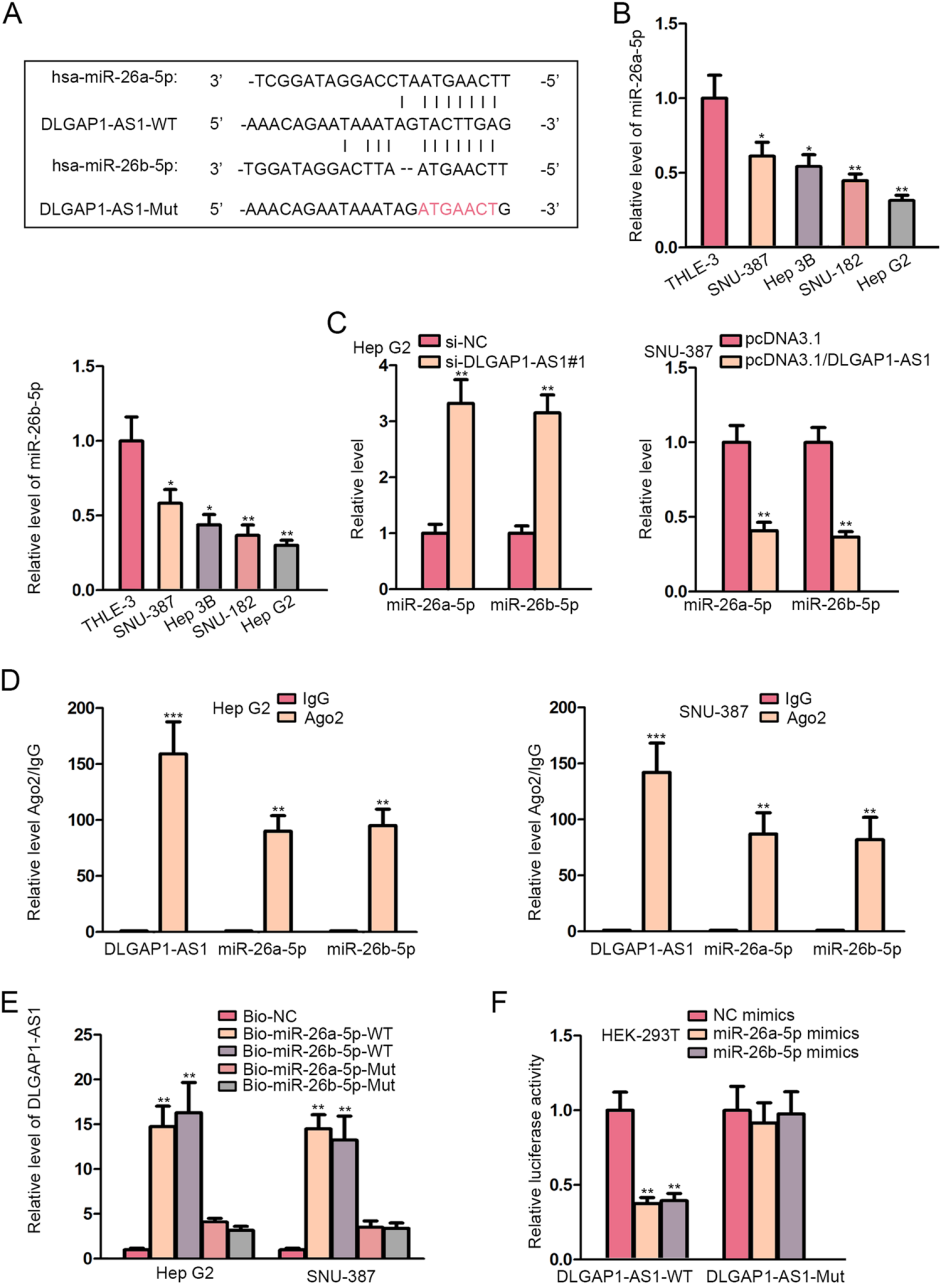
DLGAP1-AS1 acted as a molecular sponge for miR-26a-5p and miR-26b-5p. **a** The binding sites for miR-26a-5p and miR-26b-5p within DLGAP1-AS1 sequence were predicted by starBase. **b** qRT-PCR evaluated miR-26a/b-5p levels in four HCC cells and in normal cell THLE-3. **c** The effects of DLGAP1-AS1 knockdown or overexpression on miR-26a/b-5p expression were exhibited using qRT-PCR. **d** RIP assay was performed using the Ago2 antibody to demonstrate the enrichment of DLGAP1-AS1 and miR-26a/b-5p in HCC cells. **e** RNA pull-down assay was performed to detect the binding ability of DLGAP1-AS1 with miR-26a/b-5p. **f** Luciferase reporter assay was conducted in HEK-293T cells. All data are presented as the mean ± SD of three independent experiments. *p < 0.05, **p < 0.01, ***p < 0.001.

In order to examine the molecular mechanism of DLGAP1-AS1, we hypothesized that DLGAP1-AS1 might act as a ceRNA and resorted to starBase v3.0 online database to search the candidate miRNAs sequestered by DLGAP1-AS1. we found 19 miRNAs that can bind with DLGAP1-AS1. RNA pull-down assay revealed that miR-26a/b-5p showed the highest enrichment in DLGAP1-AS1-bound probe (Fig. S1a). Moreover, miR-26a-5p and miR-26b-5p, a couple of representative candidates which belong to the same miRNA family and have been occasionally investigated together, enormously attracted our interest for research. More importantly, both of them have been frequently reported to exert significant anticancerous functions on tumorigenesis and EMT in various cases of cancer, including HCC^18^. In consequence, we chose miR-26a-5p and miR-26b-5p as our objects of investigation. The binding sites within DLGAP1-AS1 sequences where they were predicted to be sponged were also illustrated (Fig. 2a).

First, opposite with DLGAP1-AS1, miR-26a-5p and miR-26b-5p were downregulated in HCC cell lines in comparison with normal cells (Fig. 2b). Besides, DLGAP1-AS1 knockdown led to elevated levels of miR-26a/b-5p in Hep G2 cells, whereas DLGAP1-AS1 overexpression resulted in miR-26a/b-5p downregulation in SNU-387 cells, implying that miR-26a/b-5p were negatively regulated by DLGAP1-AS1 (Fig. 2c). In order to determine the molecular interaction, we conducted RIP assay using antibodies against Ago2, and the enrichment of DLGAP1-AS1 and miR-26a/b-5p with Ago2 antibodies was observed, indicating that they were recruited to RNA-induced silencing complexes (RISCs) and might have functional interactions (Fig. 2d). Besides, RNA pull-down assay illustrated the direct bond between DLGAP1-AS1 and miR-26a/b-5p at the correct binding sites, because only wild-type probes for miR-26a/b-5p could significantly pull-down DLGAP1-AS1 (Fig. 2e). Moreover, DLGAP1-AS1 luciferase reporters containing wild-type and mutant binding sites were constructed and transfected into HEK-293T cells. Wild-type reporters displayed a significantly repressed luciferase activity with miR-26a/b-5p overexpression, while luciferase activity of mutant reporters could barely be lowered (Fig. 2f). In conclusion, DLGAP1-AS1 was proven to act as a molecular sponge to sequester miR-26a-5p and miR-26b-5p.

### IL-6 was targeted by miR-26a/b-5p and was under regulation of DLGAP1-AS1

**Fig. 3:**
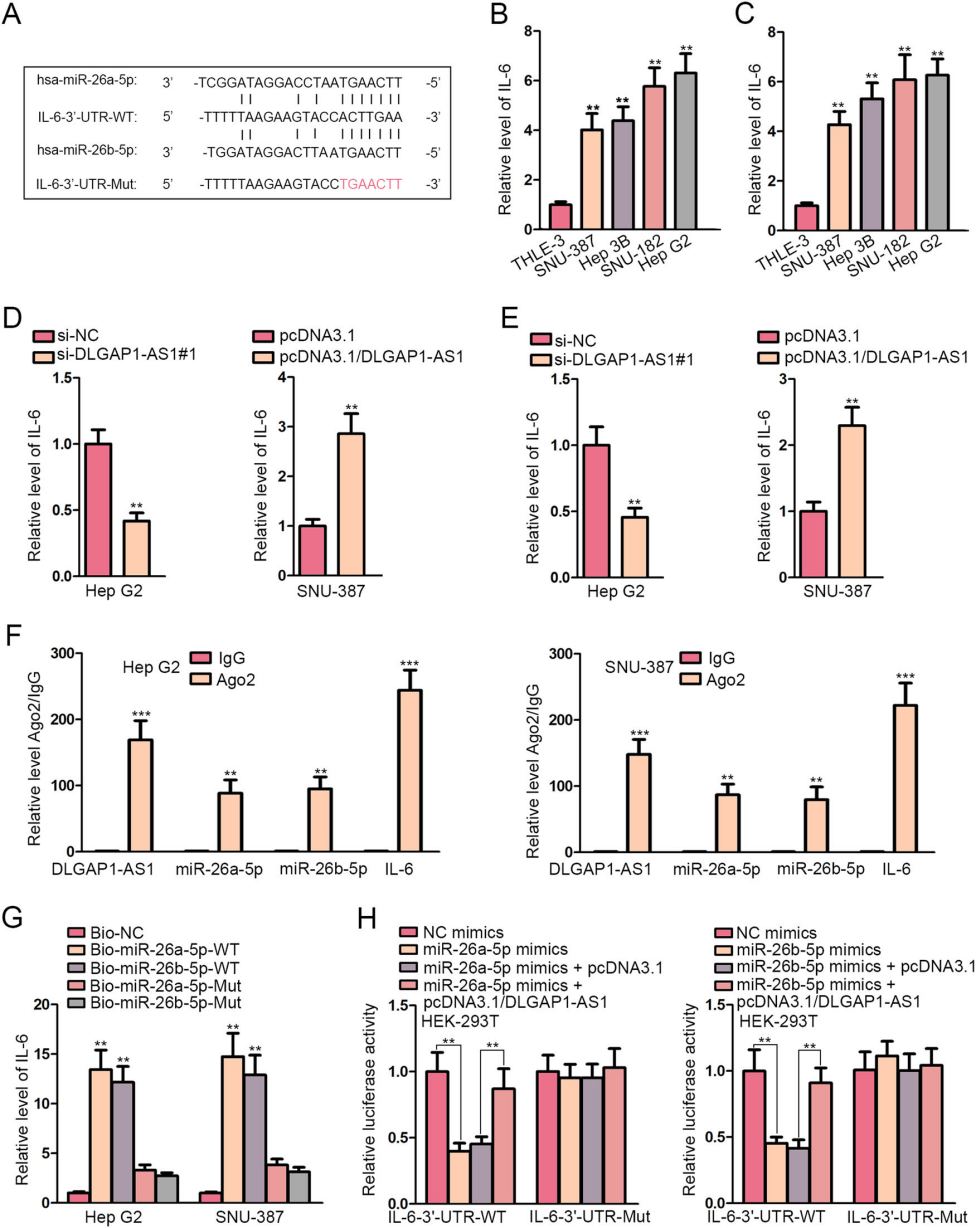
IL-6 was targeted by miR-26a/b-5p and was under regulation of DLGAP1-AS1. **a** The binding sites for miR-26a-5p and miR-26b-5p within IL-6 3′-UTR sequence was predicted by starBase. The red nucleotides represent the mutant binding site designed for luciferase reporter assay. **b** qRT-PCR evaluated IL-6 mRNA levels in HCC cell lines and in normal cells. **c** ELISA evaluated IL-6 protein levels in HCC cell lines and in normal cells. **d**, **e** The effects of DLGAP1-AS1 knockdown in Hep G2 cells and DLGAP1-AS1 overexpression in SNU-387 cells on IL-6 mRNA and protein levels were respectively exhibited using qRT-PCR and ELISA. **f** RIP assay was performed using the Ago2 antibody to demonstrate the enrichment of DLGAP1-AS1, miR-26a/b-5p and IL-6 mRNA in HCC cells. **g** RNA pull-down assay was performed to detect the binding ability of IL-6 mRNA with miR-26a/b-5p. **h** Luciferase reporter assay of IL-6-3′-UTR-WT or IL-6-3′-UTR-Mut reporters elucidated the interaction between IL-6 mRNA and miR-26a/b-5p, and the competing effect of DLGAP1-AS1 to interact with miR-26a/b-5p. All data are presented as the mean ± SD of three independent experiments. **p < 0.01, ***p < 0.001.

Based on our preceding study on the interaction between DLGAP1-AS1 and miR-26a/b-5p, we proceeded to search for potential genes targeted by miR-26a/b-5p. Using three online bioinformatics tools, we found that there were 380 mRNAs that can be regulated by both miR-26a-5p and miR-26b-5p (Fig. S1b). Next, these candidate mRNAs were subjected to qRT-PCR analysis in response to the upregulation of miR-26a-5p or miR-26b-5p. Top five downregulated mRNAs were shown in Fig. S1c, among which IL6 was expressed lowest in cells transfected with miR-26a-5p mimics or miR-26b-5p mimics. With the aid of starBase that the mRNA of IL-6, a characteristic inflammatory cytokine closely involved in cancers, was an appropriate target for them (Fig. 3a). IL-6 is noteworthy owing that it has been broadly characterized as a major cancerogenic factor contributing to malignancy, EMT and metastasis of multifarious cancers, including HCC^19^. Hence IL-6 was selected as our following study object. First, IL-6 mRNA expression was detected in HCC cell lines and normal cells, confirming its upregulation in HCC cells (Fig. 3b). Additionally, IL-6 protein level was quantified using ELISA, showing the same tendency (Fig. 3c). The influence of DLGAP1-AS1 knockdown or overexpression on IL-6 was assessed on levels of mRNA and protein, indicating IL-6 was positively related with DLGAP1-AS1, which could sponge miR-26a/b-5p and deregulate IL-6 expression (Fig. 3d, e). As for the molecular mechanism, RIP assay illustrated that DLGAP1-AS1, miR-26a/b-5p, and IL-6 mRNA were enriched in anti-Ago2 groups (Fig. 3f). RNA pull-down assay verified the binding capacity of IL-6 mRNA with wild-type biotinylated probes for miR-26a/b-5p (Fig. 3g). To determine the molecular regulation between miR-26a/b-5p and IL-6 mRNA, luciferase activity of wild-type IL-6-3′-UTR reporters was initially lowered by miR-26a/b-5p, and then partially recovered with DLGAP1-AS1. Meanwhile, the luciferase activity of mutant reporters was barely affected (Fig. 3h). In conclusion, DLGAP1-AS1 could function as a ceRNA in HCC cells to competitively bind to miR-26a-5p and miR-26b-5p, thus upregulating the downstream gene IL-6.

### The inhibitors for miR-26a/b-5p and IL-6 treatment both rescued the anti-oncogenic effects of DLGAP1-AS1 knockdown

**Fig. 4:**
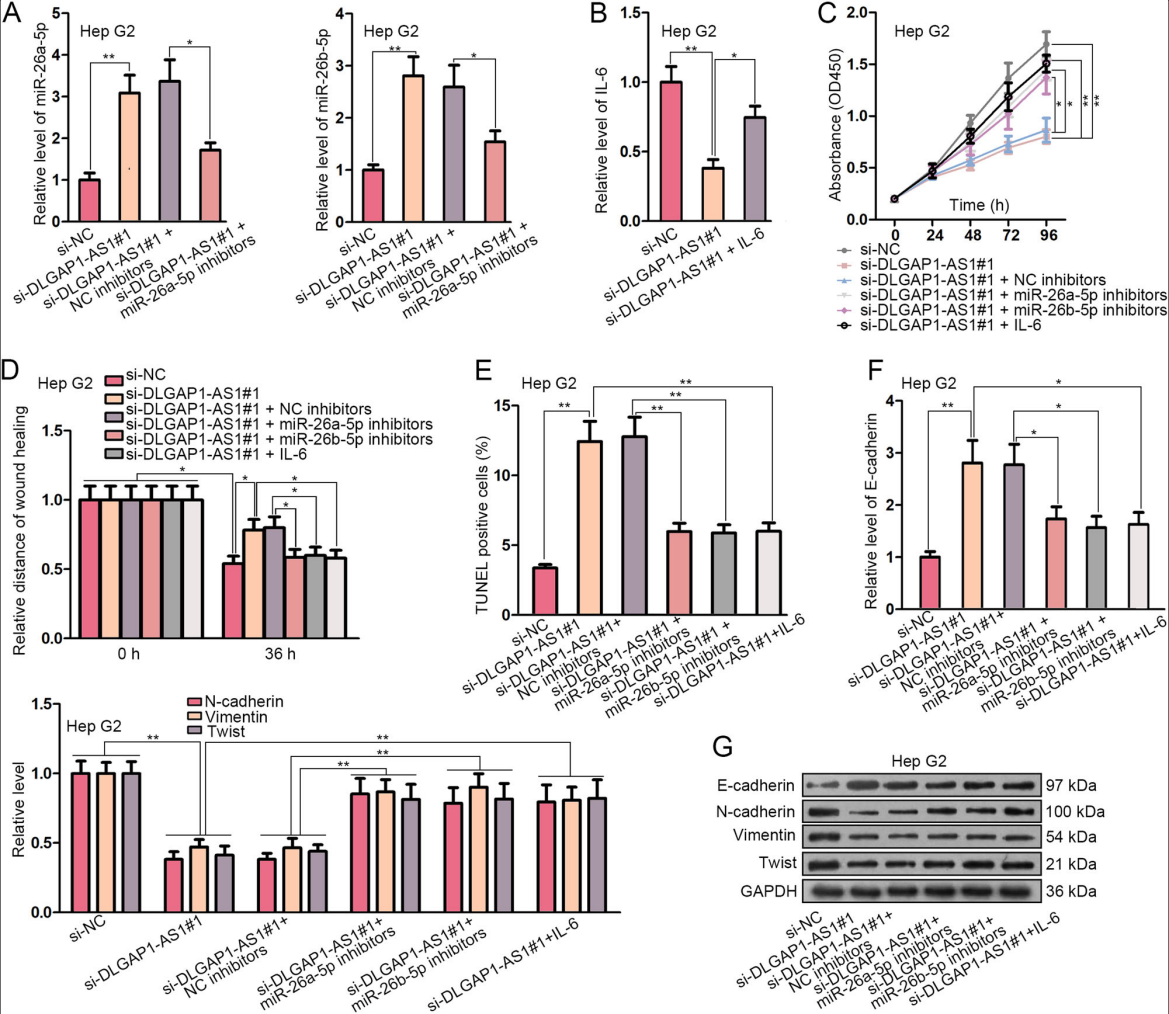
The inhibitors for miR-26a/b-5p and IL-6 treatment both rescued the anti-oncogenic effects of DLGAP1-AS1 knockdown. **a** qRT-PCR detected that miR-26a/b-5p levels in Hep G2 cells with DLGAP1-AS1 knockdown were downregulated by transfecting miR-26a/b-5p inhibitors. **b** ELISA evaluated the efficiency of IL-6 treatment in Hep G2 cells with DLGAP1-AS1 knockdown. **c** CCK-8 assay showed that both miR-26a/b-5p inhibitors and IL-6 rescued the inhibitory effect on cell proliferation of DLGAP1-AS1 knockdown. **d** Wound healing assay showed that both miR-26a/b-5p inhibitors and IL-6 rescued the inhibitory effect on cell migration of DLGAP1-AS1 knockdown. **e** TUNEL assay showed that both miR-26a/b-5p inhibitors and IL-6 rescued the promotional effect on cell apoptosis of DLGAP1-AS1 knockdown. **f**, **g** The influences of miR-26a/b-5p knockdown and IL-6 treatment on EMT-related factors in Hep G2 cells with DLGAP1-AS1 knockdown were respectively analyzed using qRT-PCR and WB. All data are presented as the mean ± SD of three independent experiments. *p < 0.05, **p < 0.01.

In order to elucidate that the biological effects of DLGAP1-AS1 were mediated through regulating miR-26a/b-5p and IL-6, we treated Hep G2 cells, which were previously transfected with si-DLGAP1-AS1#1, with miR-26a/b-5p inhibitors and human recombinant IL-6. Transfection efficiency was assessed by detecting the change of miR-26a/b-5p levels (Fig. 4a). For IL-6 treatment, the cells were incubated with 100 ng/mL of IL-6 for 48 h, and IL-6 levels were assessed using ELISA (Fig. 4b). The suppressed proliferation of Hep G2 cells with DLGAP1-AS1 knockdown was partially raised by both transfecting miR-26a/b-5p inhibitors and treating with IL-6 (Fig. 4c). The inhibited cell migration was partially enhanced by both miR-26a/b-5p inhibitors and IL-6 (Fig. 4d). Besides, cell apoptotic level initially enhanced by DLGAP1-AS1 knockdown was attenuated by both miR-26a/b-5p inhibitors and IL-6 (Fig. 4e). As for the influences on EMT-related factors, E-cadherin level enhanced by DLGAP1-AS1 knockdown was partially reduced, while N-cadherin, Vimentin and Twist levels suppressed by DLGAP1-AS1 knockdown were partially elevated (Fig. 4f, g). Similarly, we designed rescue assays in SNU-387 cells to demonstrate the DLGAP1-AS1/miR-26a/b-5p/IL6 axis. As expected, proliferation, apoptosis, migration and EMT process of SNU-387 cells that were regulated by DLGAP1-AS1 overexpression were recovered partly by overexpression of miR-26a/b-5p or silencing of IL6 (Fig. S2a–e). These results indicated that miR-26a/b-5p and IL-6 took part in the implementation of the regulatory functions of DLGAP1-AS1 in HCC cells.

### IL-6 transcriptionally elevated DLGAP1-AS1 expression in HCC cells through JAK2/STAT3 signaling pathway

**Fig. 5:**
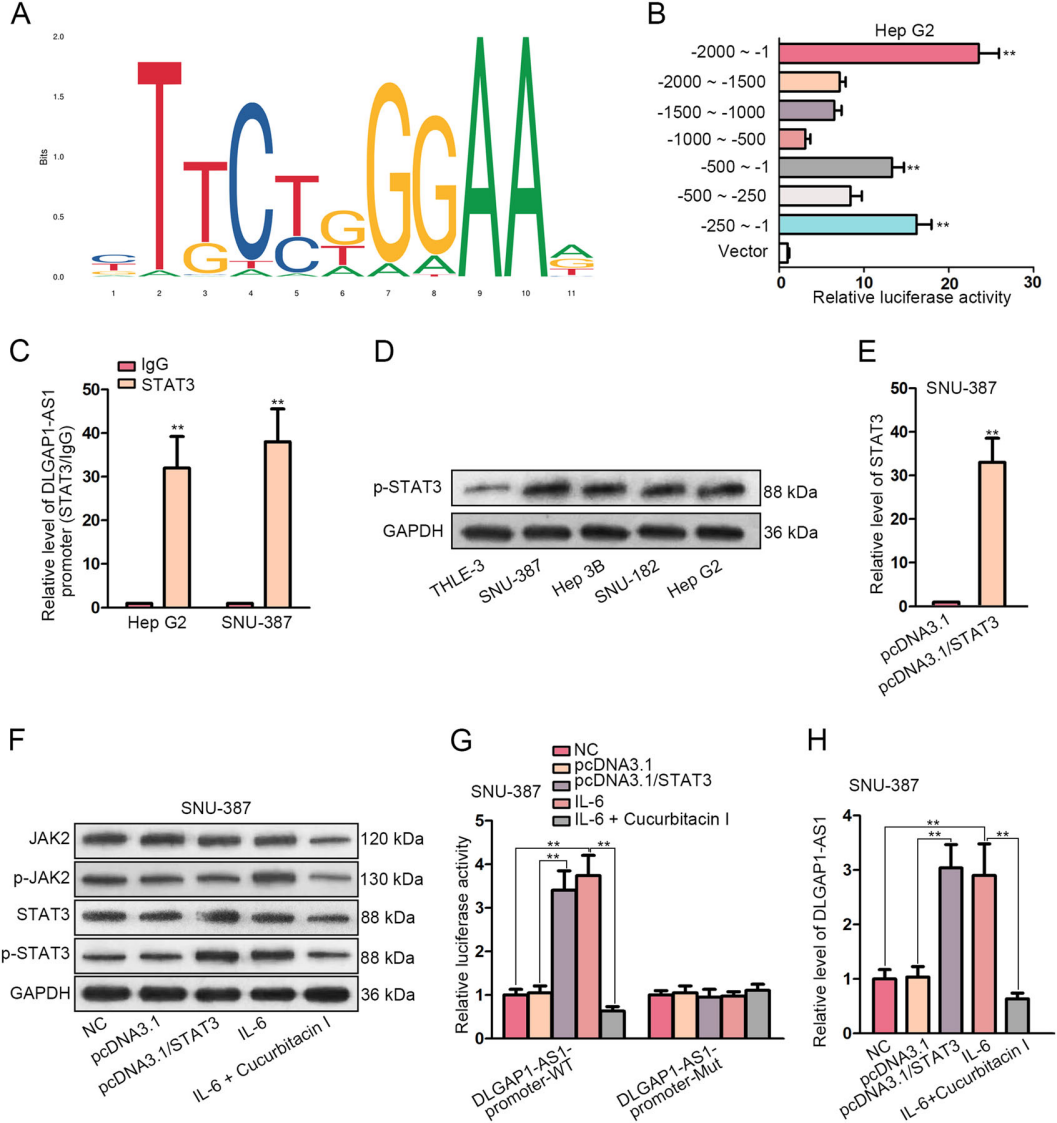
IL-6 transcriptionally elevated DLGAP1-AS1 expression in HCC cells through JAK2/STAT3 signaling pathway. **a** The STAT3 motif predicted from JASPAR database. **b** Luciferase reporter assay demonstrated luciferase activities of various truncated reporters in Hep G2 cells, determining the region of DLGAP1-AS1 promoter on which STAT3 could bind to mediate transcriptional activation. **c** ChIP assay was performed using the STAT3 antibody to demonstrate the enrichment of the STAT3-binding region of DLGAP1-AS1 promoter in Hep G2 and SNU-387 cells. **d** WB analysis evaluated phosphorylated STAT3 levels in HCC cell lines and in normal cells. (**e**) qRT-PCR evaluated the efficiency of STAT3 overexpression in SNU-387 cells. **f** WB analysis evaluated the efficiency of STAT3 overexpression and the activating effect of IL-6 on JAK2/STAT3 pathway in SNU-387 cells, while JAK2/STAT3 pathway inhibitor Cucurbitacin I was applied so that the IL-6-induced activation of JAK2 and STAT3 was reversed. **g** Luciferase reporter assay was performed in SNU-387 cells to verify that STAT3 interacted with DLGAP1-AS1 promoter at the predicted binding motif. The interaction was enhanced by IL-6 treatment and repressed by Cucurbitacin I treatment. **h** DLGAP1-AS1 expression levels influenced by STAT3, IL-6, and Cucurbitacin I were assessed using qRT-PCR. All data are presented as the mean ± SD of three independent experiments. **p < 0.01.

It is acknowledged that transcriptional activation mediated by transcription factors (TFs) or co-factors plays a critical role in contributing to the aberrant expression of cancer-related genes^20^. In order to explore the mechanism by which DLGAP1-AS1 was upregulated in HCC, we applied the online bioinformatics tools UCSC and JASPAR to examine the promoter region of DLGAP1-AS1 gene. Consequently, 17 potential binding sites for an important human TF, signal transducer and activator of transcription 3 (STAT3), were predicted within DLGAP1-AS1 promoter sequence (Fig. 5a). STAT3 is prominent as a typical transcriptional activator which plays a key role in many cancer types, such as HCC, by regulating the expression of important genes associated with cancer, thus arousing our interest^21^. In order to verify that DLGAP1-AS1 was transcriptionally under the regulation of STAT3, we constructed a variety of reporter plasmids, containing several truncations of potential region for DLGAP1-AS1 promoter (2000 bp upstream), and performed luciferase reporter assay in Hep G2 cells. We observed that higher luciferase activity was associated with the region between −500 ~ −1, while the whole sequence (−2000 ~ −1) was used as a positive control. Moreover, luciferase activity of −250 ~ -1 reporter, rather than −500 ~ −250 reporter, was elevated, suggesting that the region was most likely to be responsible for STAT3 interaction and transcriptional activation (Fig. 5b). Since the region had been shown in silico to contain one potential binding site (−73 ~ −63) for STAT3, ChIP assay using STAT3 antibodies was subsequently conducted, illustrating that the fragment of DLGAP1-AS1 promoter containing the STAT3 motif at −73 ~ −63 region was enriched in anti-STAT3 groups, further confirming the interaction between STAT3 and DLGAP1-AS1 promoter at the predicted binding site (Fig. 5c).

The levels of phosphorylated STAT3 (p-STAT3, the activated form) were detected in HCC and normal cells, illustrating that STAT3 activation was promoted in HCC cells, to which the elevated DLGAP1-AS1 level could be attributed (Fig. 5d). Intriguingly, it has been elucidated that STAT3 can be activated by IL-6 through Janus kinase 2 (JAK2), and IL-6/JAK2/STAT3 has been implied as a crucial accelerator for tumorigenesis and EMT in many cancer types, including HCC^22^. Since IL-6 had been proven to be positively regulated by DLGAP1-AS1, we wondered whether DLGAP1-AS1 could be reciprocally upregulated by IL-6 through activating JAK2 and STAT3. Therefore, we established STAT3 overexpression model in SNU-387 cells where DLGAP1-AS1 expression was relatively moderate (Fig. 5e). We performed WB analysis in SNU-387 cells, and found that STAT3 and p-STAT3 levels were enhanced by pcDNA3.1/STAT3, implying the overexpression efficiency. Furthermore, phosphorylated JAK2 (p-JAK2, the activated form) and p-STAT3 were upregulated after IL-6 treatment. Nevertheless, a supplement of 0.5 μM Cucurbitacin I, a specific inhibitor of JAK2/STAT3 pathway, notably attenuated the expression and activation of JAK2 and STAT3. These results verified that IL-6 could activate STAT3 via JAK2 (Fig. 5f).

Subsequently, we performed luciferase reporter assay using DLGAP1-AS1-promoter reporters containing wild-type and mutant STAT3 motifs. STAT3 overexpression or IL-6 treatment significantly elevated luciferase activity of wild-type reporters, and Cucurbitacin I reversed the promotional effect of IL-6 on luciferase activity. However, luciferase activity of mutant reporters could scarcely be affected. These results suggested that IL-6 could enhance the transcriptional activity of DLGAP1-AS1 through facilitating the interaction between STAT3 and DLGAP1-AS1 promoter at the predicted motif (Fig. 5g). Additionally, DLGAP1-AS1 expression was enhanced by STAT3 overexpression or IL-6 treatment, and reduced by Cucurbitacin I supplementation, exhibiting the same tendency as luciferase reporter assay did (Fig. 5h). Altogether, IL-6 could reciprocally elevate DLGAP1-AS1 transcription via JAK2/STAT3 signaling pathway, thus forming a positive feedback loop for enhancing DLGAP1-AS1 expression.

### CDK8 and LRP6 were targeted by miR-26a/b-5p and were under regulation of DLGAP1-AS1

**Fig. 6:**
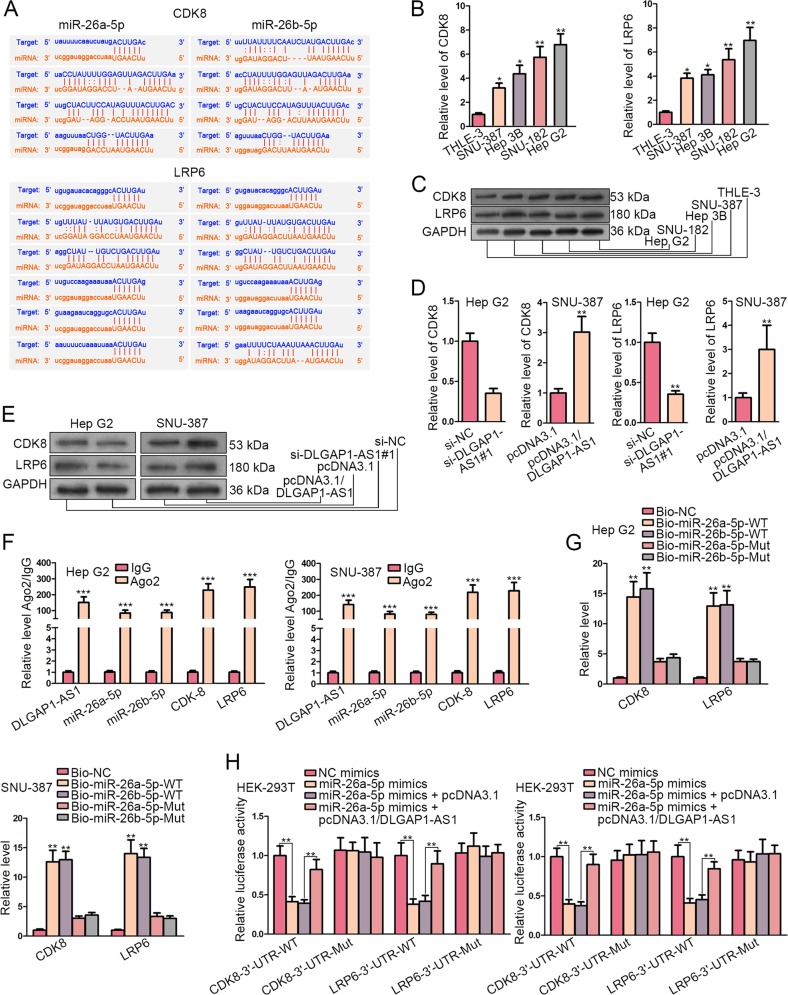
CDK8 and LRP6 were targeted by miR-26a/b-5p and were under regulation of DLGAP1-AS1. **a** The binding sites for miR-26a-5p (left) and miR-26b-5p (right) within the 3′-UTR sequences of CDK8 (top) and LRP6 (bottom) were exhibited through starBase prediction. **b** qRT-PCR evaluated CDK8 and LRP6 mRNA levels in HCC cell lines and in normal cells. **c** WB analysis evaluated CDK8 and LRP6 protein levels in HCC cell lines and in normal cells. **d**, **e** The effects of DLGAP1-AS1 knockdown in Hep G2 cells and DLGAP1-AS1 overexpression in SNU-387 cells on CDK8 or LRP6 expression were exhibited using qRT-PCR and WB. **f** RIP assay was performed using the Ago2 antibody to demonstrate the enrichment of DLGAP1-AS1, miR-26a/b-5p and mRNAs of CDK8 and LRP6 in HCC cells. **g** RNA pull-down assay was performed to detect the binding ability of CDK8 or LRP6 mRNA with miR-26a/b-5p. **h** Luciferase reporter assay elucidated the interaction between CDK8 or LRP6 mRNA and miR-26a/b-5p, and the competing effect of DLGAP1-AS1 to interact with miR-26a/b-5p. All data are presented as the mean ± SD of three independent experiments. *p < 0.05, **p < 0.01, ***p < 0.001.

Considering the partial rescue of IL6 for DLGAP1-AS1 in HCC cells, we further investigated whether some other downstream targets exerted functions in DLGAP1-AS1-induced HCC cell activities. Our research continued to pursue potential downstream genes that could participate in hepatocarcinogenesis via activating Wnt/β-catenin pathway. Then, we analyzed whether DLGAP1-AS1 and miR-26a/b-5p could regulate the activity of Wnt/β-catenin pathway by directly regulating CTNNB1. Through luciferase reporter assays, we determined that DLGAP1-AS1 and miR-26a/b-5p could not directly regulate CTNNB1 (Fig. S3a–c), thus to activating Wnt/β-catenin pathway. With the help of starBase, we discovered the binding sequences between miR-26a/b-5p and the 3′-UTR regions of cyclin-dependent kinase 8 (CDK8) and low density lipoprotein receptor-related protein 6 (LRP6) (Fig. 6a). CDK8 has been identified to be a hallmark regulator to activate Wnt/β-catenin signaling through β-catenin stabilization^23^. LRP6 has been recognized as a co-receptor to facilitate Wnt/β-catenin signaling via promoting β-catenin nuclear translocation^24^. Besides, both CDK8 and LRP6 have been reported to act as oncogenes in HCC^25^. As a consequence, these two genes were chosen as our study objects. The expression levels of CDK8 and LRP6 were evaluated likewise using qRT-PCR for mRNAs and WB for proteins, illustrating their increase in HCC cells compared with normal cells (Fig. 6b, c). Besides, CDK8 and LRP6 were positively regulated by DLGAP1-AS1 on mRNA and protein levels (Fig. 6d, e). To demonstrate the molecular mechanism, the enrichment of DLGAP1-AS1, miR-26a/b-5p and mRNAs of CDK8 and LRP6 in anti-Ago2 groups was exhibited by RIP assay, indicating the recruitment of these molecules in RISCs (Fig. 6f). RNA pull-down assay showed that wild-type miR-26a/b-5p probes could significantly pull-down mRNAs of CDK8 or LRP6, illustrating their binding capacity (Fig. 6g). Moreover, luciferase activity of wild-type CDK8 or LRP6-3′-UTR reporters, not of mutant reporters, was reduced by miR-26a/b-5p, and partially enhanced by addition of pcDNA3.1/DLGAP1-AS1 (Fig. 6h). In conclusion, DLGAP1-AS1 could also act as a ceRNA to sponge miR-26a/b-5p and regulate CDK8 and LRP6.

### DLGAP1-AS1 promotes HCC development and EMT via Wnt/β-catenin pathway activation through CDK8 and LRP6

**Fig. 7:**
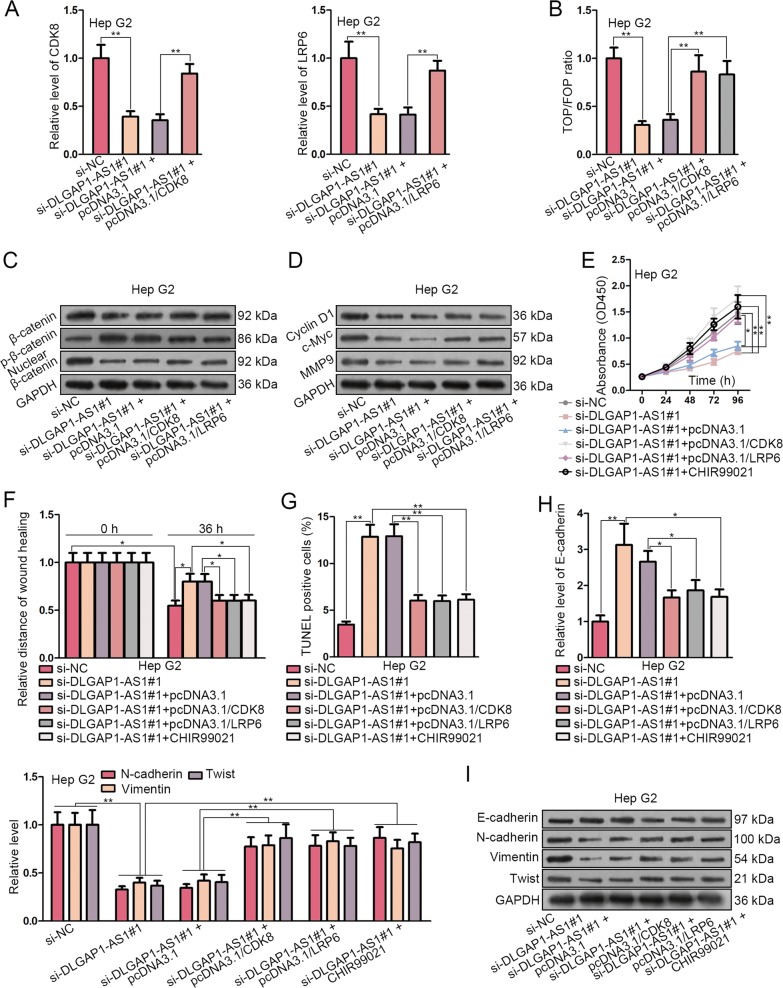
DLGAP1-AS1 promotes HCC development and EMT via Wnt/β-catenin pathway activation through CDK8 and LRP6. **a** qRT-PCR detected that CDK8 or LRP6 level in Hep G2 cells with DLGAP1-AS1 knockdown was upregulated by transfection of pcDNA3.1/CDK8 or pcDNA3.1/LRP6. **b** TOP/FOP flash assay was performed to verify the deactivating effect of DLGAP1-AS1 knockdown on TCF/LEF transcription, which was reactivated by CDK8 or LRP6 overexpression. **c** The influences of DLGAP1-AS1 knockdown and CDK8 or LRP6 overexpression on β-catenin expression, phosphorylation, and nuclear translocation were evaluated using WB analysis. **d** WB analysis displayed the protein levels of several typical downstream genes of Wnt/β-catenin pathway, which were downregulated by DLGAP1-AS1 knockdown and then upregulated by transfection of pcDNA3.1/CDK8 or pcDNA3.1/LRP6. **e**, **f** CCK-8 assay and wound healing assay showed that the inhibitory effects on cell proliferation and migration of DLGAP1-AS1 knockdown were attenuated by overexpression of CDK8 and LRP6, as well as by treatment with CHIR99021. **g** TUNEL assay showed that the promotional effect on cell apoptosis of DLGAP1-AS1 knockdown was attenuated by CDK8, LRP6 or CHIR99021. **h**, **i** The influences of CDK8 and LRP6 overexpression and CHIR99021 treatment on EMT-related factors in Hep G2 cells with DLGAP1-AS1 knockdown were respectively analyzed using qRT-PCR and WB. All data are presented as the mean ± SD of three independent experiments. *p < 0.05, **p < 0.01.

We subsequently discussed the involvement of CDK8 and LRP6 in facilitating HCC progression and EMT via activating Wnt/β-catenin pathway. First, CDK8 or LRP6 overexpression plasmids were transfected into Hep G2 cells which had been transfected with DLGAP1-AS1 siRNAs previously. The transfection efficiency was evaluated by detecting the variation of CDK8 or LRP6 expression (Fig. 7a). Next, TOP/FOP flash assay was conducted to detect the degree of β-catenin-mediated T-cell factor/lymphoid enhancer factor (TCF/LEF) transcriptional activation. The result illustrated the inhibitory effect of DLGAP1-AS1 knockdown, and the promotional effect of CDK8 or LRP6 overexpression on Wnt/β-catenin pathway activity (Fig. 7b). It was also illustrated that DLGAP1-AS1 knockdown reduced total protein level of β-catenin, facilitated β-catenin phosphorylation (a label for degradation), and inhibited β-catenin nuclear translocation, while CDK8 or LRP6 overexpression could partially rescue these effects (Fig. 7c). Furthermore, several typical downstream genes of Wnt/β-catenin pathway, namely Cyclin D1, c-Myc and MMP9, were downregulated by DLGAP1-AS1 knockdown, and partially upregulated by CDK8 or LRP6 overexpression (Fig. 7d). These results demonstrated that DLGAP1-AS1 could positively regulate Wnt/β-catenin pathway activity via CDK8 and LRP6. Then we evaluated how the properties of DLGAP1-AS1-silenced Hep G2 cells were affected by CDK8 or LRP6 overexpression, or by treatment of 6 μM CHIR99021, a typical Wnt/β-catenin pathway activator. As a result, cell proliferation and migration suppressed by DLGAP1-AS1 knockdown were partially reversed (Fig. 7e, f), cell apoptosis enhanced by DLGAP1-AS1 knockdown was partially attenuated (Fig. 7g), the enhanced level of E-cadherin was downregulated, and the suppressed levels of N-cadherin, Vimentin, and Twist were upregulated (Fig. 7h, i). In summary, our results demonstrated that DLGAP1-AS1 could up-regulate CDK8 and LRP6 to activate Wnt/β-catenin pathway in HCC cells, thus promoting tumorigenesis and EMT.

### DLGAP1-AS1 contributed to HCC growth and metastasis in vivo

**Fig. 8:**
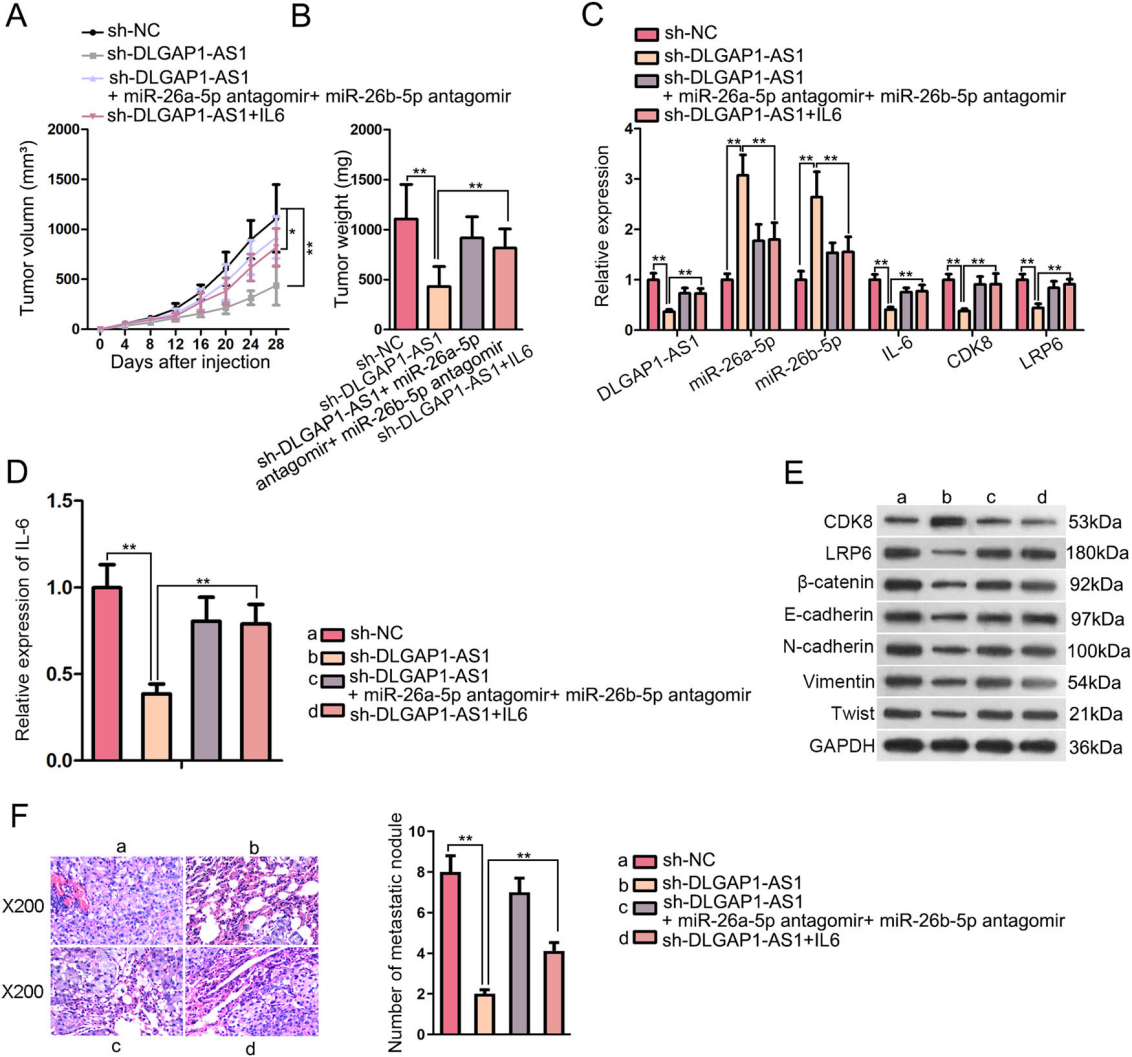
DLGAP1-AS1 contributed to HCC growth and metastasis in vivo. **a** Tumor volume from different treatment groups was measured after injection. **b** Tumor weight from different treatment groups was measured after the mice were euthanized. **c**–**e** The expression levels of several genes related with the present study from each group of xenograft were evaluated using qRT-PCR, ELISA and WB. **f** Representative images of HE-stained mouse lung tissues were taken to demonstrate lung metastasis of HCC xenograft. The amount of metastatic nodules from each group was calculated and analyzed accordingly. All data are presented as the mean ± SD of three independent experiments. *p < 0.05, **p < 0.01.

We further investigated the contribution of DLGAP1-AS1 to promoting HCC growth and metastasis by adopting an in vivo tumor model. After the xenograft tumor model had been established, compared with sh-NC group, tumors with significantly smaller size and lighter weight were developed in sh-DLGAP1-AS1 group, while miR-26a/b-5p suppression or IL6 overexpression rescued the inhibitory effect of DLGAP1-AS1 knockdown on tumorigenicity in vivo (Fig. S4a and Fig. 8a, b). Furthermore, the expression levels of genes involved in our study were measured using qRT-PCR, ELISA and WB from xenograft tumor tissues, showing that the expression tendencies of these genes were in consistence with those in vitro (Fig. 8c–e). Eventually, we evaluated the capacity of tumor metastasis through observing and measuring the metastatic nodules transferring to lung tissues, illustrating that HCC lung metastasis was prominently inhibited by DLGAP1-AS1 knockdown, and the inhibitory effect could be reversed by knockdown of miR-26a/b-5p or the upregulation of IL6 (Fig. 8f). Moreover, SNU-387 cells transfected with pcDNA3.1, pcDNA3.1/DLGAP1-AS1, pcDNA3.1/DLGAP1-AS1, pcDNA3.1/DLGAP1-AS1+miR-26a/b-5p antagomir or pcDNA3.1/DLGAP1-AS1+sh-IL6 were injected into the nude mice. Afterward, we observed that the tumor growth and metastasis were promoted by the upregulation of DLGAP1-AS1, while were inhibited after overexpression of miR-26a/b-5p or silencing of IL6 (Fig. S4b–d). To be concluded, our results validated the cancerogenic function of DLGAP1-AS1 in vivo.

Next, clinical significance of DLGAP1-AS1/miR-26a/b-5p/IL6 axis was analyzed in HCC patients. At first, the expression of DLGAP1-AS1 was elevated in HCC samples compared to adjacent normal samples (Fig. S5a). In addition, high expression level of DLGAP1-AS1 was observed in tissues collected from patients with metastasis and recurrence (Fig. S5a). After grouping the patients into two groups (high and low) in accordance with the mean value of DLGAP1-AS1 expression, Kaplan–Meier analysis was made. The results showed that patients in high expression group had a poorer prognosis than those in low expression group (Fig. S5b). Furthermore, the low expression of miR-26a/b-p and high expression of IL6 were assessed in HCC tissues compared with adjacent normal tissues (Fig. S5c, d). Accordingly, Pearson correlation test showed that miR-26a/b-5p was negatively correlated with DLGAP1-AS1 or IL6 (Fig. S5e, f), whereas the DLGAP1-AS1 and IL6 were positively correlated with each other (Fig. S5g).

## Discussion

HCC remains a major health issue worldwide with increasing occurrence and poor prognosis. The majority of HCC cases take place in developing countries, among which China is one of the most high-risk areas worldwide^26^. EMT is a complicated biological process involving many regulating elements and signaling pathways. Our research herein discussed a potential mechanism that could facilitate EMT capacity of HCC cells.

Since numerous studies have reported that lncRNAs can exert their regulatory functions in HCC progression in a miRNA-dependent pattern to protect target mRNAs from being degraded^27,28^, we assumed DLGAP1-AS1 to act as a ceRNA, and found using starBase that DLGAP1-AS1 sequence contained the binding sites for miR-26a-5p and miR-26b-5p, whose lowered expression in HCC cells and downregulation by DLGAP1-AS1 were illustrated. RIP, RNA pull-down and luciferase reporter assays were subsequently conducted, thus verifying the prediction that DLGAP1-AS1 directly interacted with miR-26a/b-5p. Both miR-26a-5p and miR-26b-5p have been reported as HCC suppressors^18,29^. Moreover, miR-26a-5p and miR-26b-5p together have been reported to be able to inhibit carcinogenesis and metastasis in many cancers, such as oral squamous cell carcinoma^30^, prostate cancer^31^ and bladder cancer^32^. Therefore, the involvement of miR-26a/b-5p in DLGAP1-AS1-induced biological effects was explored.

IL-6 is an inflammatory cytokine with multiple essential physiological and pathological functions. Autocrine, paracrine or circulating IL-6 acting on cancer cells has long been regarded as a major oncogenic factor^33^. In the present study, IL-6 was proven to be targeted by miR-26a/b-5p through bioinformatics analysis and detecting molecular interaction. Besides, IL-6 was upregulated and under the regulation of DLGAP1-AS1 in HCC cells. Furthermore, the influences of DLGAP1-AS1 knockdown on proliferation, migration, apoptosis and EMT-related factors were rescued by overexpression of miR-26a/b-5p inhibitors or IL-6 treatment, indicating the ceRNA network concerning DLGAP1-AS1, miR-26a/b-5p and IL-6 in HCC cells.

Subsequently, we investigated the potential transcription activator responsible for DLGAP1-AS1 upregulation. With the help of bioinformatics tools, we found that the motif of STAT3, which is noteworthy as a key regulator for transcription of various cancer-related genes^21^, existed within DLGAP1-AS1 promoter sequence, indicating that STAT3 could be a potential TF for DLGAP1-AS1. Subsequently, luciferase reporter assay and ChIP assay illustrated the molecular interaction of STAT3 and DLGAP1-AS1 promoter at the predicted binding site. The level of activated STAT3 was enhanced in HCC cell lines, consistent with that of DLGAP1-AS1. IL-6, as a cytokine, is capable to act on cancer cells to activate JAK-STAT pathway, thus inducing carcinogenic effects such as proliferation, apoptosis inhibition, metastasis, and angiogenesis^34^. Here, we hypothesized that IL-6 could reciprocally promote DLGAP1-AS1 transcriptional via activating JAK2 and STAT3. Our results elucidated that IL-6 enhanced the levels of activated JAK2 and STAT3, and both STAT3 overexpression and IL-6 treatment elevated the transcriptional activity and the expression level of DLGAP1-AS1. Additionally, these effects were reversed by addition of JAK2/STAT3 pathway inhibitor Cucurbitacin I. The feedback loop by which DLGAP1-AS1 expression was enhanced in return was therefore discovered.

Wnt/β-catenin pathway, also known as the canonical Wnt pathway, is a highly conserved signaling pathway whose activation has an association with multiple cancer types, including HCC^7^. Besides, many crucial genes related with cancer progression, such as Cyclin D1, c-Myc, and MMP9, are modulated by Wnt/β-catenin pathway. Herein, our research continued to pursue potential downstream genes participating in hepatocarcinogenesis via activating Wnt/β-catenin pathway. We found through bioinformatics analysis, RIP, RNA pull-down and luciferase reporter assays that CDK8 and LRP6, both of which have been proven as oncogenes in HCC and able to activate Wnt/β-catenin pathway^25^, were targeted and regulated by miR-26a/b-5p. CDK8 and LRP6 were also upregulated and under the regulation of DLGAP1-AS1 in HCC cells. Furthermore, the biological functions of DLGAP1-AS1 knockdown were partially reversed by CDK8 or LRP6 overexpression, or by addition of Wnt/β-catenin pathway activator CHIR99021, indicating the ceRNA network concerning DLGAP1-AS1, miR-26a/b-5p, and CDK8/LRP6 to activate Wnt/β-catenin pathway. Finally, the in vivo experiments on xenograft models further verified the cancerogenic effect of DLGAP1-AS1 on tumor growth and metastasis of HCC, suggesting the potential clinical value of DLGAP1-AS1.

In conclusion, the present study demonstrated that DLGAP1-AS1 facilitated HCC tumorigenesis and EMT by sponging miR-26a-5p and miR-26b-5p in vitro and in vivo. The participation of IL-6/JAK2/STAT3 pathway and Wnt/β-catenin pathway was also demonstrated to play important roles in mediating the oncogenic function of DLGAP1-AS1. Our results suggest the potentiality of DLGAP1-AS1 as a biomarker for HCC treatment, and provide a new insight for understanding the molecular mechanisms associated with HCC.

## Supplementary information

Supplementary data

## References

[1] Bray F (2018). Global cancer statistics 2018: GLOBOCAN estimates of incidence and mortality worldwide for 36 cancers in 185 countries. CA Cancer J. Clin..

[2] Forner A, Reig M, Bruix J (2018). Hepatocellular carcinoma. Lancet.

[3] Portolani N (2006). Early and late recurrence after liver resection for hepatocellular carcinoma: prognostic and therapeutic implications. Ann. Surg..

[4] Ye LY (2016). Hypoxia-induced epithelial-to-mesenchymal transition in hepatocellular carcinoma induces an immunosuppressive tumor microenvironment to promote metastasis. Cancer Res..

[5] Kalluri R, Weinberg RA (2009). The basics of epithelial-mesenchymal transition. J. Clin. Invest.

[6] Kang FB (2015). B7-H3 promotes aggression and invasion of hepatocellular carcinoma by targeting epithelial-to-mesenchymal transition via JAK2/STAT3/Slug signaling pathway. Cancer Cell Int.

[7] Zhang Q (2013). Wnt/beta-catenin signaling enhances hypoxia-induced epithelial-mesenchymal transition in hepatocellular carcinoma via crosstalk with hif-1alpha signaling. Carcinogenesis.

[8] Ponting CP, Oliver PL, Reik W (2009). Evolution and functions of long noncoding RNAs. Cell.

[9] Kopp F, Mendell JT (2018). Functional classification and experimental dissection of long noncoding RNAs. Cell.

[10] Wahlestedt C (2013). Targeting long non-coding RNA to therapeutically upregulate gene expression. Nat. Rev. Drug Disco..

[11] Wong CM, Tsang FH, Ng IO (2018). Non-coding RNAs in hepatocellular carcinoma: molecular functions and pathological implications. Nat. Rev. Gastroenterol. Hepatol..

[12] Ding CH (2018). The HNF1alpha-regulated lncRNA HNF1A-AS1 reverses the malignancy of hepatocellular carcinoma by enhancing the phosphatase activity of SHP-1. Mol. Cancer.

[13] Huang JL (2018). The long non-coding RNA PTTG3P promotes cell growth and metastasis via up-regulating PTTG1 and activating PI3K/AKT signaling in hepatocellular carcinoma. Mol. Cancer.

[14] Xiao Y (2016). MiR-503 inhibits hepatocellular carcinoma cell growth via inhibition of insulin-like growth factor 1 receptor. Onco Targets Ther..

[15] Yao J (2012). GNAI1 suppresses tumor cell migration and invasion and is post-transcriptionally regulated by Mir-320a/c/d in hepatocellular carcinoma. Cancer Biol. Med.

[16] Li YL (2019). BAY 87-2243 sensitizes hepatocellular carcinoma Hep3B cells to histone deacetylase inhibitors treatment via GSK-3beta activation. Exp. Ther. Med.

[17] Feng X (2019). CRABP2 regulates invasion and metastasis of breast cancer through hippo pathway dependent on ER status. J. Exp. Clin. Cancer Res.

[18] Shen G (2014). MicroRNA-26b inhibits epithelial-mesenchymal transition in hepatocellular carcinoma by targeting USP9X. BMC Cancer.

[19] Shintani Y (2016). IL-6 secreted from cancer-associated fibroblasts mediates chemoresistance in NSCLC by increasing epithelial-mesenchymal transition signaling. J. Thorac. Oncol..

[20] Malz M, Pinna F, Schirmacher P, Breuhahn K (2012). Transcriptional regulators in hepatocarcinogenesis–key integrators of malignant transformation. J. Hepatol..

[21] Chen ZZ (2016). LncSox4 promotes the self-renewal of liver tumour-initiating cells through Stat3-mediated Sox4 expression. Nat. Commun..

[22] Singh AK (2018). Novel Indole-fused benzo-oxazepines (IFBOs) inhibit invasion of hepatocellular carcinoma by targeting IL-6 mediated JAK2/STAT3 oncogenic signals. Sci. Rep..

[23] Firestein R (2008). CDK8 is a colorectal cancer oncogene that regulates beta-catenin activity. Nature.

[24] Tung EK, Wong BY, Yau TO, Ng IO (2012). Upregulation of the Wnt co-receptor LRP6 promotes hepatocarcinogenesis and enhances cell invasion. PLoS One.

[25] Zeng XC (2014). Downregulation of miR-610 promotes proliferation and tumorigenicity and activates Wnt/beta-catenin signaling in human hepatocellular carcinoma. Mol. Cancer.

[26] Ferlay J (2015). Cancer incidence and mortality worldwide: sources, methods and major patterns in GLOBOCAN 2012. Int J. Cancer.

[27] Wang Y (2017). Long non-coding RNA CASC2 suppresses epithelial-mesenchymal transition of hepatocellular carcinoma cells through CASC2/miR-367/FBXW7 axis. Mol. Cancer.

[28] Lin YH (2018). Taurine up-regulated gene 1 functions as a master regulator to coordinate glycolysis and metastasis in hepatocellular carcinoma. Hepatology.

[29] Yang X (2013). MicroRNA-26a suppresses tumor growth and metastasis of human hepatocellular carcinoma by targeting interleukin-6-Stat3 pathway. Hepatology.

[30] Fukumoto I (2015). MicroRNA expression signature of oral squamous cell carcinoma: functional role of microRNA-26a/b in the modulation of novel cancer pathways. Br. J. Cancer.

[31] Kato M (2015). MicroRNA-26a/b directly regulate La-related protein 1 and inhibit cancer cell invasion in prostate cancer. Int J. Oncol..

[32] Miyamoto K (2016). Tumour-suppressive miRNA-26a-5p and miR-26b-5p inhibit cell aggressiveness by regulating PLOD2 in bladder cancer. Br. J. Cancer.

[33] Sansone P (2007). IL-6 triggers malignant features in mammospheres from human ductal breast carcinoma and normal mammary gland. J. Clin. Invest.

[34] Yu H, Kortylewski M, Pardoll D (2007). Crosstalk between cancer and immune cells: role of STAT3 in the tumour microenvironment. Nat. Rev. Immunol..

