# Supplementary material for: Long non-coding RNA DLGAP1-AS1 facilitates tumorigenesis and epithelial–mesenchymal transition in hepatocellular carcinoma via the feedback loop of miR-26a/b-5p/IL-6/JAK2/STAT3 and Wnt/β-catenin pathway
Source: Cell Death Dis. 2020 Jan 16;11(1):34. doi: 10.1038/s41419-019-2188-7 (PMC6965175; doi:10.1038/s41419-019-2188-7)
Supplement: Supplementary file 1 — Supplementary data [file 41419_2019_2188_MOESM1_ESM.docx]

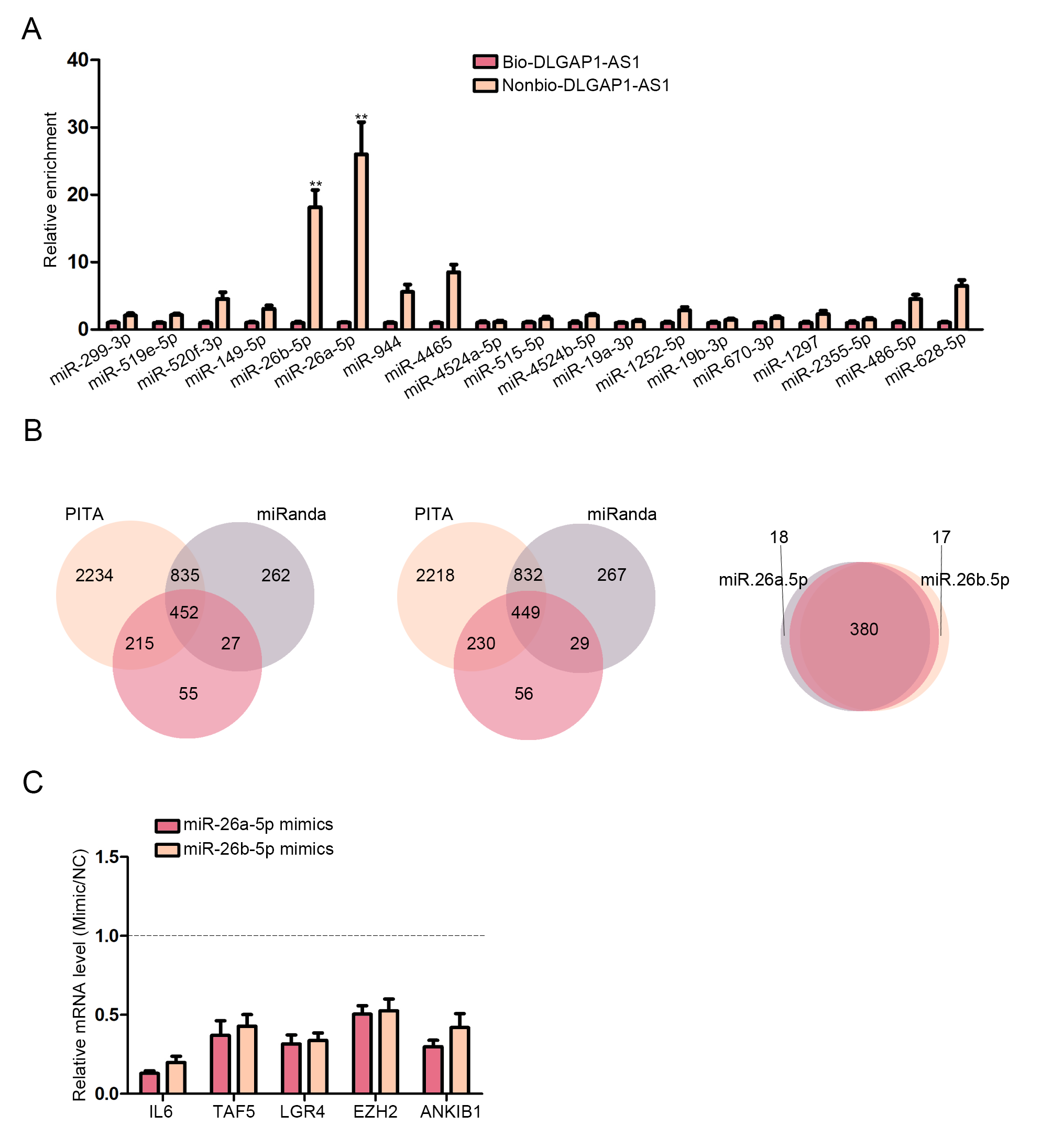


**Figure S1** (A) RNA pull-down assay was carried out to search out miRNAs that can interact with DLGAP1-AS1. (B) Venn plot showed the predicted targets of both miR-26a-5p and miR-26b-5p from three bioinformatics websites. (C) Top 5 mRNAs that were downregulated in response to the upregulation of miR-26a-5p or miR-26b-5p was detected by qRT-PCR. **p < 0.01.


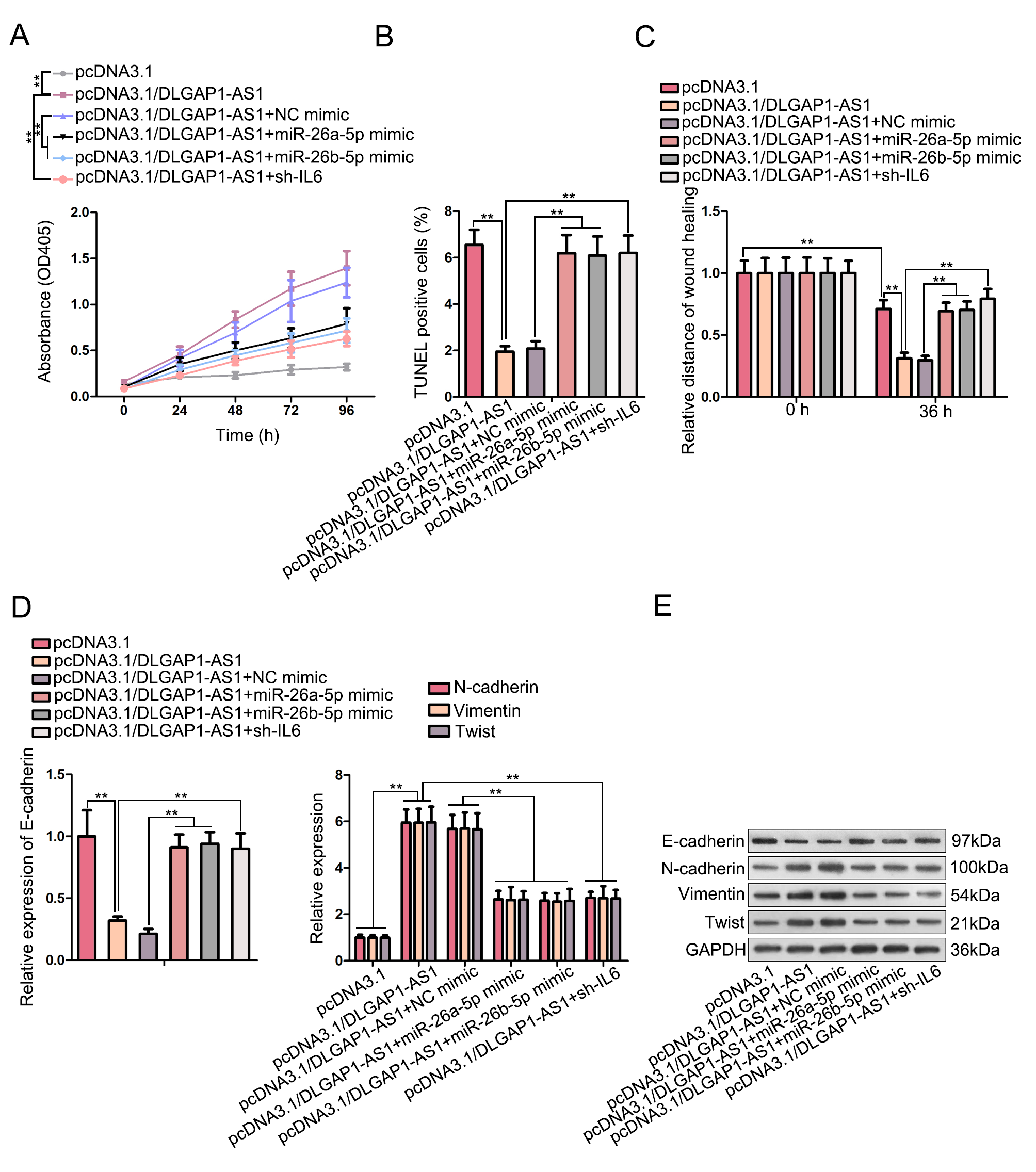


**Figure S2** Functional rescue assays were conducted in SNU-387 ells with transfection of pcDNA3.1, pcDNA3.1/DLGAP1-AS1, pcDNA3.1/DLGAP1-AS1+NC mimics, pcDNA3.1/DLGAP1-AS1 + miR-26a-5p mimics, pcDNA3.1/DLGAP1-AS1 + miR-26b-5p mimics, pcDNA3.1/DLGAP1-AS1 + sh-IL6. (A) CCK-8 assay showed the changes in cell viability after different transfections. (B) TUNEL assay showed that both miR-26a/b-5p mimics and sh-IL-6 rescued the promotional effect of DLGAP1-AS1 overexpression on cell apoptosis. (C) Wound healing assay showed that both miR-26a/b-5p mimics and sh-IL-6 rescued the inhibitory effect on cell migration of DLGAP1-AS1 overexpression. (D-E) The influences of miR-26a/b-5p mimics and sh-IL-6 on EMT-related factors in SNU-387 cells with DLGAP1-AS1 overexpression were respectively analyzed using qRT-PCR and WB. All data are presented as the mean ± SD of three independent experiments. **p < 0.01.


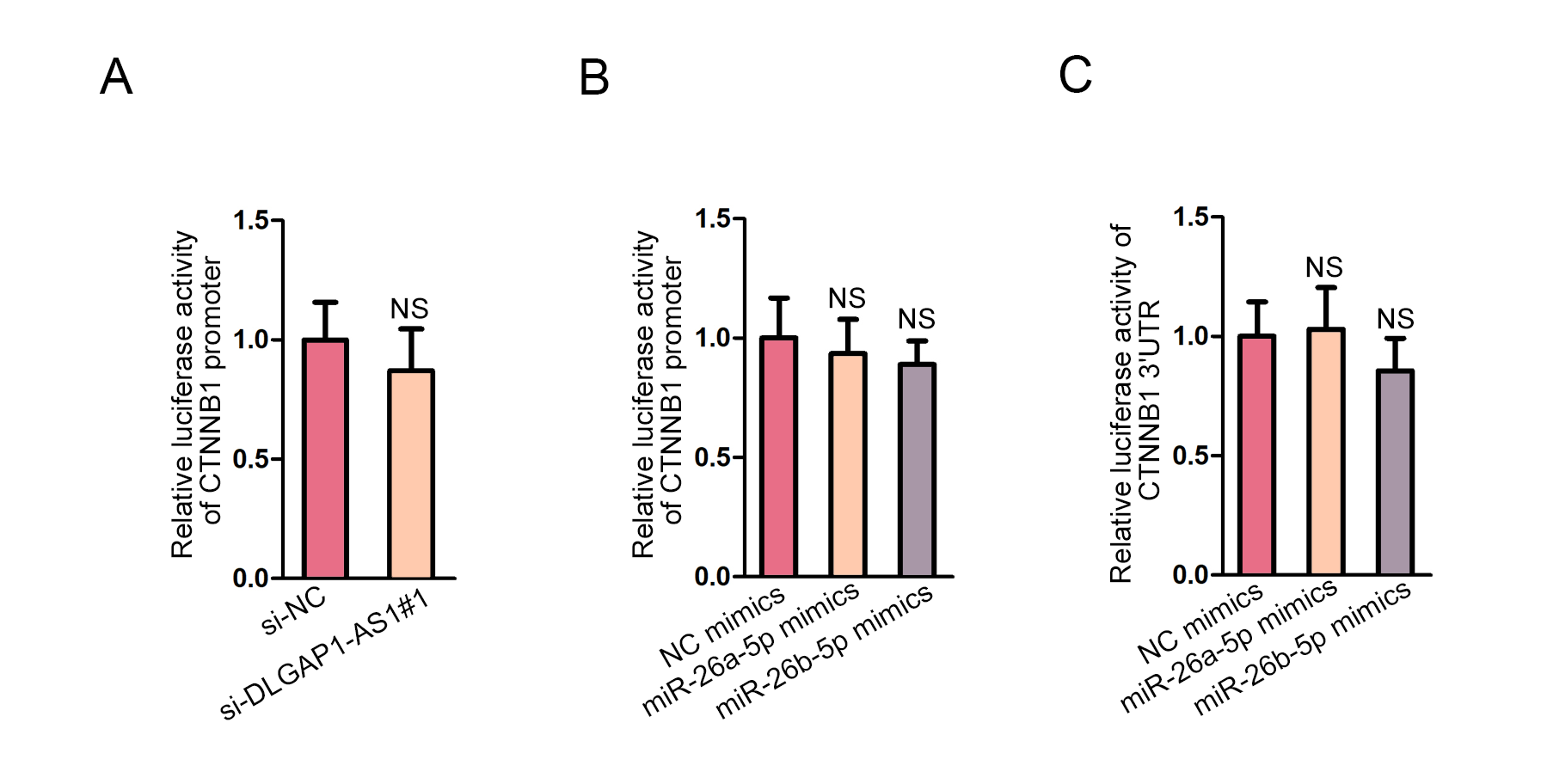


**Figure S3** (A-C) Luciferase activity analyses were carried out to evaluate the effect of DLGAP1-AS1 or miR-26a/b-5p on the CTNNB1 promoter activity and the activity of CTNNB1 3’UTR. All data are presented as the mean ± SD of three independent experiments. N.S: no significance.


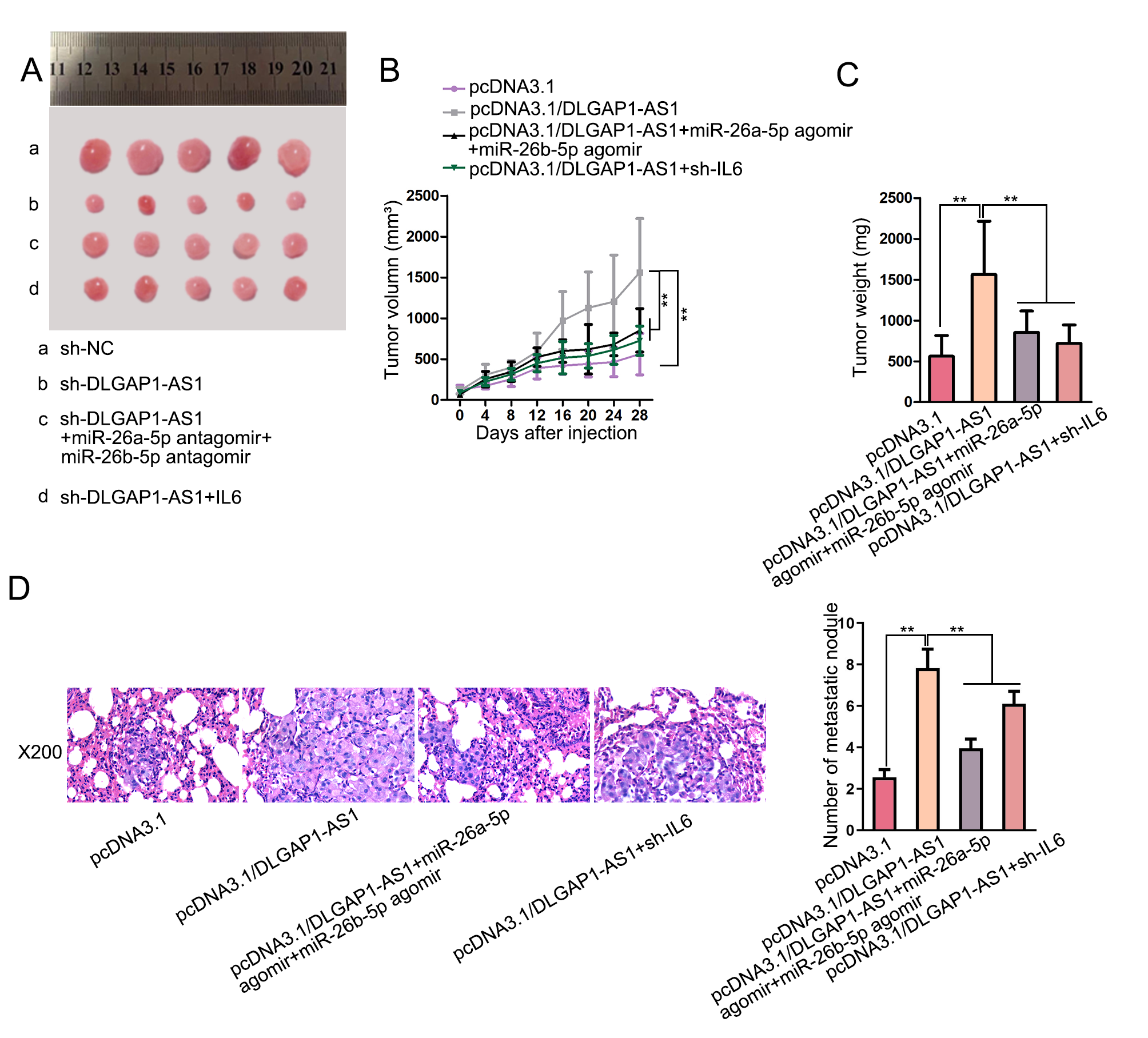


**Figure S4** (A) Tumors in consistent with Fig. 8A. (B) Tumor growth conditions in nude mice injected with transfected SNU-387 cells were observed. (C) Metastasis nodules in lung were observed in nude mice that were tail vein injected with transfected SNU-387 cells. All data are presented as the mean ± SD of three independent experiments. **p < 0.01.


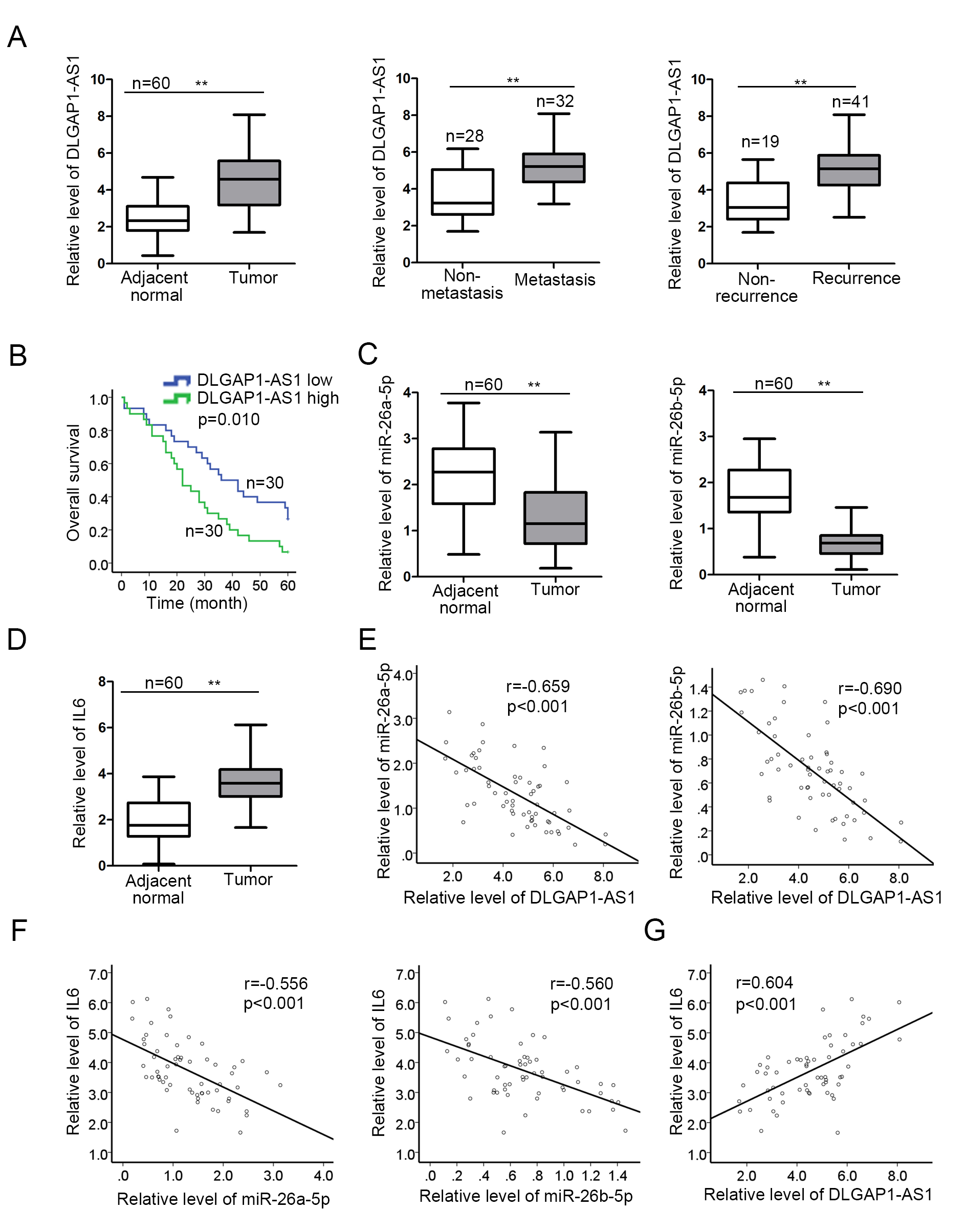


**Figure S5** (A) DLGAP1-AS1 expression in different kinds of HCC tissues. (D) Overall survival of HCC patients with high or low level of DLGAP1-AS1. (C-D) The expression levels of miR-26a/b-5p or IL6 in HCC tissues or adjacent normal tissues. (E-G) Expression correlations among DLGAP1-AS1, miR-26a/b-5p or IL6 in HCC tissues. All data are presented as the mean ± SD of three independent experiments. **p < 0.01.
